# Nanoparticles and convergence of artificial intelligence for targeted drug delivery for cancer therapy: Current progress and challenges

**DOI:** 10.3389/fmedt.2022.1067144

**Published:** 2023-01-06

**Authors:** Kaushik Pratim Das, Chandra J

**Affiliations:** Department of Computer Science, Christ University, Bangalore, India

**Keywords:** nanoparticles, artificial intelligence, biomarker, drug delivery, cancer, cancer therapy

## Abstract

Cancer is a life-threatening disease, resulting in nearly 10 million deaths worldwide. There are various causes of cancer, and the prognostic information varies in each patient because of unique molecular signatures in the human body. However, genetic heterogeneity occurs due to different cancer types and changes in the neoplasms, which complicates the diagnosis and treatment. Targeted drug delivery is considered a pivotal contributor to precision medicine for cancer treatments as this method helps deliver medication to patients by systematically increasing the drug concentration on the targeted body parts. In such cases, nanoparticle-mediated drug delivery and the integration of artificial intelligence (AI) can help bridge the gap and enhance localized drug delivery systems capable of biomarker sensing. Diagnostic assays using nanoparticles (NPs) enable biomarker identification by accumulating in the specific cancer sites and ensuring accurate drug delivery planning. Integrating NPs for cancer targeting and AI can help devise sophisticated systems that further classify cancer types and understand complex disease patterns. Advanced AI algorithms can also help in biomarker detection, predicting different NP interactions of the targeted drug, and evaluating drug efficacy. Considering the advantages of the convergence of NPs and AI for targeted drug delivery, there has been significantly limited research focusing on the specific research theme, with most of the research being proposed on AI and drug discovery. Thus, the study's primary objective is to highlight the recent advances in drug delivery using NPs, and their impact on personalized treatment plans for cancer patients. In addition, a focal point of the study is also to highlight how integrating AI, and NPs can help address some of the existing challenges in drug delivery by conducting a collective survey.

## Introduction

Cancer is a generic term for a broad category of disease that occurs due to the transformation of normal cells into tumor cells comprising of multi-stage progress from cancerous lesions to malignancy. Over a million cancer incidences are reported yearly, leading to high mortality rates (1). Conventional cancer treatment involves surgical procedures for cancer in a localized stage, followed by radiation therapy and chemotherapy for advanced stages of cancer (2). In retrospect, it has been revealed that chemotherapeutic drugs target cancer cells and specific normal cells in patients. Therefore, a new generation of cancer treatments has evolved over the past years, such as targeted cancer therapies for more precision in cancer treatment. Targeted therapies include pharmacological agents that are administered to inhibit cancer cell growth and death, leading to restricting cancer metastasis. However, recent researchers indicate that systemic drug delivery administration is a leading cause of clinical failures associated with chemotherapy due to insufficient drug concentration in the tumor regions (2).

Recently, nanomedicine and nano-delivery systems have emerged as a means for localized drug delivery for targeted tumor sites and assisting as a diagnostic tool (3). Nanotechnology plays a vital role in developing modern drug delivery systems as natural compounds are now being investigated for treating cancer and several other microbial and inflammatory diseases (4, 5). Employing nanotechnology enables the application of nanostructures and other curative agents developed at a nanoscale level for nanomedicine. The field of biomedicine encompasses nanobiotechnology, drug delivery, biosensors, and tissue engineering, which are significantly influenced by the use of NPs (6). In the past decade, several advances have been witnessed in nanotechnology, and possible fabrication, characterization, and modifications of NPs functional properties are now implemented for medical diagnosis and biomedical applications (7). NPs comprise materials designed at an atomic and molecular level, resulting in smaller nanospheres. Hence, nanoscale-sized particles can navigate freely in the human body compared to more extensive materials (8).

Conventional chemotherapy has been considered successful to a certain extent, but the significant drawbacks of chemotherapy are poor bioavailability, high-dosage requirements and adverse side effects post-therapy, lower level of therapeutic indices, chemotherapy resistance, and non-specific targeting (9–11). As the field of nanomedicine continues to make substantial strides, researchers have discussed the effectiveness of NPs-mediated drug delivery and its ability to enhance the localized drug delivery system. The key benefit of such targeted drug delivery is reducing the frequency of cancer patient dosages and any side effects. Traditionally, a direct route for administering chemo-drugs has been the preferred approach. However, drug encapsulation in a drug carrier offers several advantages, such as protecting from degrading in the bloodstream, offering better drug solubility, decreasing toxic side effects, and improving pharmacokinetic and pharmacodynamics drug properties and better drug efficacy (9). Consequently, several innovative and novel methods are now deployed in drug delivery for cancer treatments. Various types of NPs, such as metal, organic, polymeric NPs, and liposomes, are evaluated for targeted drug delivery, as some drugs tend to have poor solubility and absorption (12).

**Figure 1:**
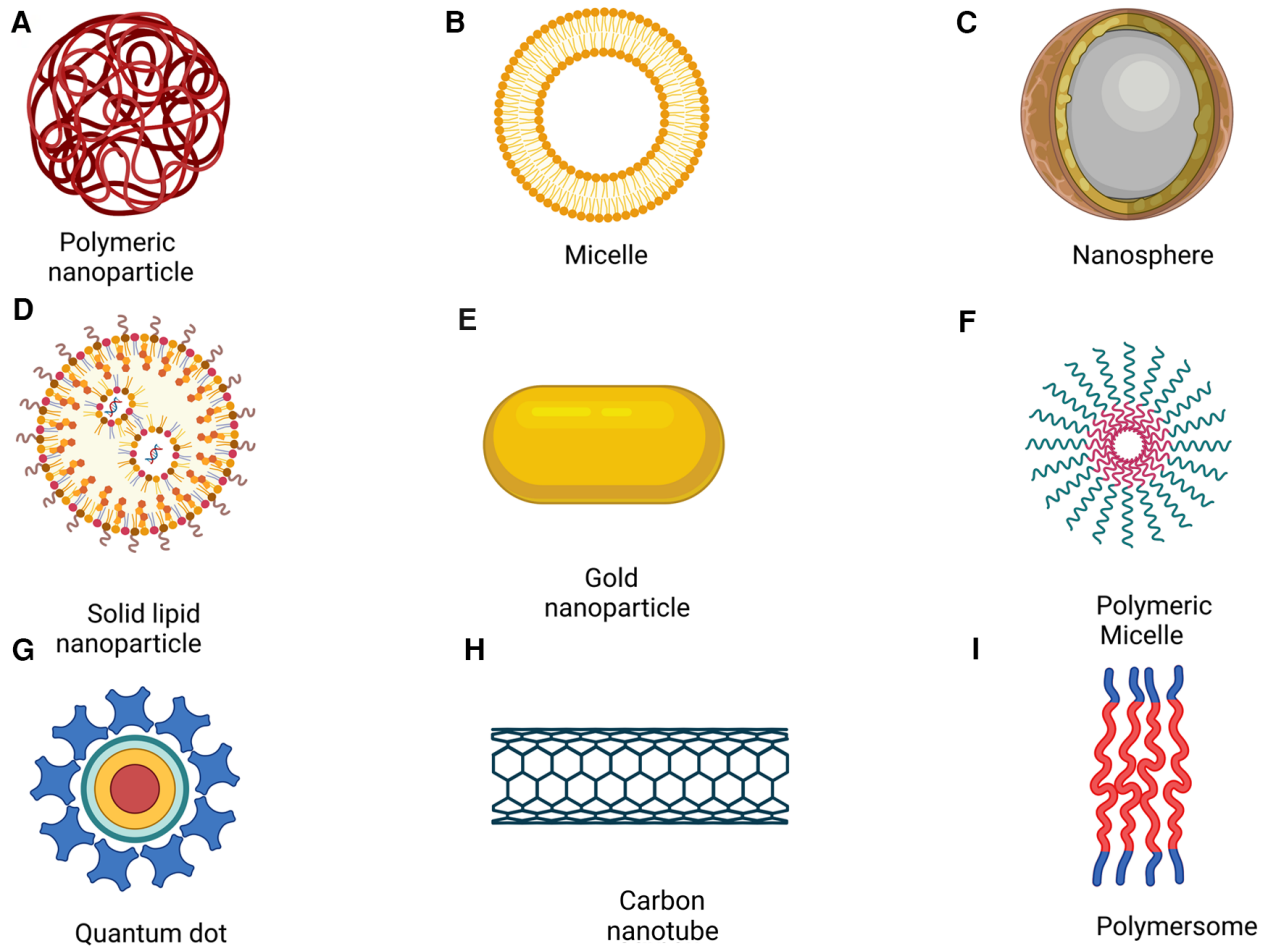
Nanoparticles for drug delivery. (Reprinted from “Nanoparticle-Mediated Targeted Drug Delivery to Cancer Stem Cells”, by BioRender, June 2020).

Cancer treatment is challenging as there are various cancer biomarkers, and each patient has a distinct molecular profile. This diversity is apparent in different cancer types as patients have unique molecular signatures and distinct driver mutations leading to tumor heterogeneity which is a critical challenge in cancer treatment (13, 14). More recently, diagnostic NPs ranging from quantum dots (QDs), gold NPs (AuNPs) to polymer dots (PDs) are investigated to create patient-specific disease profiles, which can be further leveraged using therapeutic nanotechnologies to help realize precision medicine and improve personalized patient treatment outcomes (15, 16) (see Figure 1). However, due to patient tumor heterogeneity, it is challenging to rationally build a diagnostic and therapeutic platform that can accurately analyze the output. In this case, the convergence of artificial intelligence (AI) and nanotechnologies is a promising approach as AI can provide rapid analysis of large amounts of patient data, predict disease progression, evaluate pharmacological profiles, and cancer biomarker detection as potential avenues (15). Moreover, AI is highly effective in optimizing; therefore, nanomedicine may also benefit from AI integration by optimizing material properties or understanding targeted drugs and their interactions with the immune system or cell membranes faster and with drug synergies (15).

The advances in AI and its potential contribution to bionanotechnology present a unique opportunity to realize its full potential in precision medicine for cancer diagnosis and treatment. This is because AI has immense capabilities for automation and faster patient analysis of complex disease information by faster processing of complex medical data and delivering accurate results, thereby improving treatment outcomes. This review highlights how AI can help rationally transform clinical information into actionable insights for cancer therapy. In addition, the study emphasizes that AI may be used for biomarker detection to enhance targeted drug delivery systems. Ultimately, the findings may serve as foundations for overcoming challenges associated with low response rates and clinical trial failures, understanding drug synergies and as additional tools for analysis and molecular docking to facilitate computer and AI-enabled drug design procedures, and contributing towards more affordable cancer treatments using AI and nanomedicines.

## Background

### Nanotechnology for drug delivery and precision medicine

Nanotechnology is an emerging field that fuses science and technology to develop diverse biomedical and manufacturing engineering applications, where materials are developed at the nanometer scale. These nanosized materials have a significant advantage over bulk materials regarding better surface-to-volume ratio. Recently, NPs have been used for various applications such as sensing, actuating, agriculture, biomedical analysis, and medicine. In medicine, NPs play a pivotal role in developing advanced solutions for drug delivery, cancer screening, and tissue engineering (17, 18). NPs have been identified to effectively deliver recombinant proteins, nucleotides, and vaccines (19). When considering drug delivery and medical imaging applications for cancer diagnosis and treatment, a broad category of NPs is explored as they provide the feasibility to be synthesized using both organic and inorganic materials ranging from lipids, proteins, polymers, natural compounds, metals, and carbon-based nanomaterials (15, 20). However, different types of NPs used for targeted drug delivery have advantages and disadvantages.

Recent efforts have been directed at investigating and improving nanotechnology in medicine, of which NPs-based studies significantly contribute to its advancement. As new research and development initiatives focus on optimizing drug delivery platforms, engineered NPs are investigated as potential solutions. Various studies reveal their effectiveness and ability to overcome conventional delivery limitations, such as bio-distribution, cell-specific targeting, and molecular transportation to desired organelles (21). In addition, NPs are considered to enhance the stability and solubility of drug-encapsulated cargos to help promote better transportation across cell membranes with prolonged drug circulation time in patients to increase drug efficacy and safety (22). However, despite widespread research on NPs, the implementation of NPs-mediated drug delivery to patients is drastically low due to the lack of understanding of the physiological and pathological differences in animal and human studies. This gap in the studies limits the understanding of the functionality and behavior of the human body and nanomedicine. Further, the heterogeneity in patients is also a critical factor that limits the success of nanomedicine, specifically in treating complex diseases like cancer (23, 24).

However, more recent studies have explored how NPs can overcome biological barriers and increase the precision with more personalized treatment approaches by utilizing patient information such as genetic, environmental, and historical factors, including biomarker information, to develop individualized treatment plans. Lipids, polymers, and inorganic NPs are continuously investigated using different approaches towards their synthesis to help optimize drug delivery leading to the success of precision medicine in cancer treatment. However, precision therapies may also be subject to biological barriers that can be overcome with the help of greater utilization of accurate data from a stratified patient population that can improve the response to precision medicine therapies, enhance drug specificity, and optimizes drug dose delivery (21).

### Targeted drug delivery methods for cancer treatment

**Figure 2:**
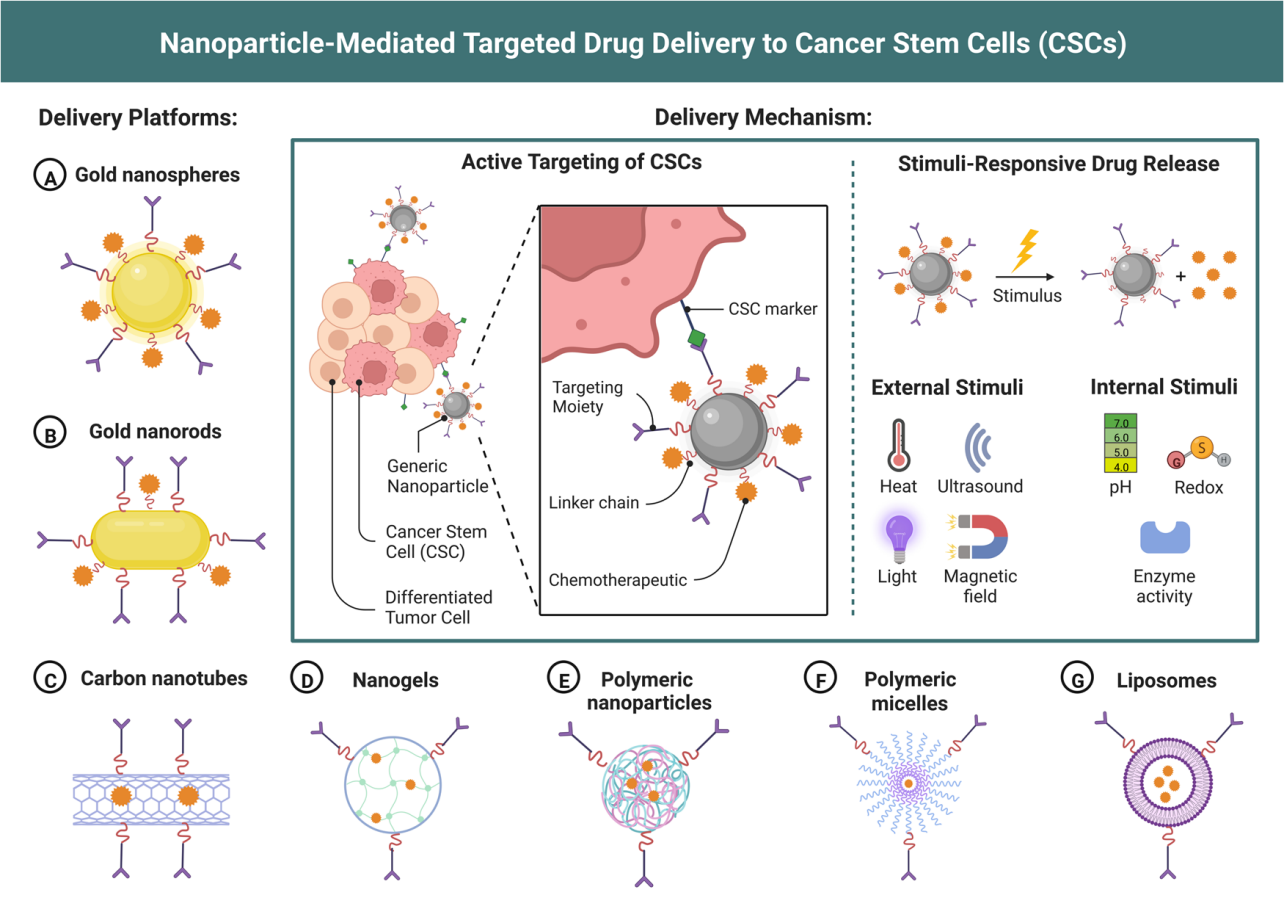
Nanoparticle-Mediated targeted drug delivery for cancer treatment. (Reprinted from “Nanoparticle-Mediated Targeted Drug Delivery to Cancer Stem Cells”, by BioRender, June 2020).

Over the past decades, there have been several advances and successes in cancer treatments, primarily due to an improved understanding of carcinogenesis processes, cell biology, and tumor microenvironment (25). However, cancer is a complex disease; therefore, many types of cancer still have a high fatality rate. Targeted drug delivery in this context is highly critical for improving the survival rates of cancer patients with information that can ensure the accurate delivery of anticancer drugs (26). In addition, drug targeting helps to define the selective release of cancer drugs at the specific tumor site with a higher pharmacological impact. Two targeting methods can be achieved using NPs: active and passive (27). In passive targeting, localization of NPs is best achieved for the organ of interest within the tumor microenvironment, whereas active targeting allows for identifying the uptake levels of NPs by the tumor cells (28) (see Figure 2).

Recent advances have enabled personalized treatments for individual patients, where the targeted cancer therapies are categorized as monoclonal antibodies, small molecule inhibitors, and immunotoxins (29). Targeted cancer therapies aim to address some of the challenges of conventional chemotherapy, where the targeted drugs impact the rapidly developing cancer cells and specific normal cells such as intestinal epithelium. In this regard, targeted drug delivery focuses on using pharmacological agents that inhibit cell growth and cell death, thereby restricting cancer metastasis. In addition, rather than focusing on molecular changes, targeted cancer therapies interfere with the protein interactions responsible for tumorigenesis (29).

The enhanced permeability and retention (EPR) effect is one of the primary considerations for effective drug delivery to tumor tissues. The EPR effect is a unique feature associated with tumor cells that are considered a significant milestone in tumor targeting with chemotherapy. Therefore, this EPR effect has become essential in anticancer drug development and various drug design and delivery strategies using macromolecule agents, molecular imaging, antibody therapy, liposomes, and protein-polymer conjugates, respectively (17, 30). However, there are serious disputes about the impact of EPR when using NPs (31). Thus, there is an urgent need for increased attention toward developing tumor-specific delivery systems for anticancer agents, and as such, recognizing the intrinsic differences between normal and tumor cells is crucial for better drug efficacies (32).

Nanotechnology is vital in developing targeted drug delivery systems and improving human therapeutics. Like any new advances, drug delivery systems using NPs is at a beginning state but with a promising future. Novel nanotechnology approaches are continuously investigated to enhance drug delivery; however, there are still a significant amount of challenges, such as (a) limited knowledge about NPs components and characteristics, (b) lack of uniformity of toxicity, (c) lacking standardized model systems and assays, (d) non-availability of standard protocols for synthesis; (e) lacking in efficient and advanced analytical tools; (f) gaps in understanding how NPs may impact or interact with biological systems; (g) unavailability of *in vivo* monitoring systems, and (h) no standardized safety guidelines (33). Thus, artificial intelligence has great potential to address some of these challenges and improve targeted drug delivery.

#### Existing drug delivery systems for cancer treatment

The food and Administration (FDA) have been continuously involved with developing NP-based drug delivery systems for the past several years. For example, an albumin-based nanoparticle formulation consisting of paclitaxel is observed in Abraxane, which has achieved commercial success in treating breast cancer (34, 35). At the same time, the current drug delivery systems mainly implement synthetic polymers such as poly D, L-lactic-co-glycolic acid (PLGA) because of biodegradability and biocompatibility (36). Intralipid is an FDA-approved emulsion injection comprising soybean oil and water formulations and is stabilized with the help of an egg phospholipid emulsifier. This formulation successfully addressed the challenges of solubility, buffering, passive targeting, and stability (34, 37).

Similarly, a significant obstacle is observed for nanodrugs based cancer therapy due to high uptakes in the reticuloendothelial system, resulting in a lesser impact of the nano-drug on the tumor site and increasing toxicity. Consequently, Intralipid has been found to improve bioavailability and reduce cytotoxicity in monocytic cells. In addition, a combination of Intralipid and Abraxane has been identified to reduce tumor growth significantly (38). In recent years, polymer-based drug delivery systems have been studied for drug delivery and ensuring the controlled release of anticancer agents with consistent doses over long periods. Furthermore, with advancements in nanotechnology, polymeric nanoparticles are studied as potential drug delivery systems for cancer treatments due to their physiochemical properties and the ability to improve tumor localization (39). Recently, Vyxeos, an FDA-approved combination chemotherapy nanoparticle that Jazz Pharmaceuticals developed, has been targeted for treating acute myeloid leukemia (40). Since 2016, various clinical trials of the drug have shown that Vyexos is successful in improving drug efficacy even at lower concentrations of dose than free drug administration.

Moreover, it has been identified that a combination of daunorubicin and cytarabine can be delivered using the Vyexos drug delivery system in synergetic ratios for cancer treatment. As a result, improved interaction with the target cell is observed upon drug release (40). At the same time, Myocet liposomal is developed by Teva, UK, and approved by the European Medicines Agency (EMA), which is composed of liposomal doxorubicin (non-PEGylated) that is primarily administered for treating breast cancer metastasis (41, 42). In addition, Nanobiotix has developed NBTXR3, a radiohancer consisting of hafnium oxide nanoparticles capable of eradicating tumors during radiotherapy. Clinical trials suggest that when NBTXR3 is activated, it absorbs more energy from the radiotherapy and improves the dose delivered, resulting in higher possibilities of tumor cell death without damaging the healthy tissues (40, 43, 44). Several advances and more nanoparticle-based drug-delivery systems are under clinical trials, and a significant number have also been approved by the FDA and EMA, respectively. Therefore, it is evident that there will be several new additions in future studies upon successful clinical trials and approval.

## Methods

The general PRISMA methodology for literature review has been adopted to find the answers to our review's formulated research questions (45). The key publications are selected based on the literature search in scientific databases, including computer science, biomedical and biotechnology journals and conferences such as IEEE, SAGE Journals, Springer, Elsevier, MDPI, and Frontiers. In addition, biomedical literature is searched from the National Library of Medicine, mainly from National Centre for Biotechnology Information. The study focused on identifying relevant research; hence, research themes such as (Drug Delivery and Drug Design), (NPs for Targeted Drug Delivery), (Artificial Intelligence for Drug Delivery), (Artificial Intelligence and Bionanotechnology) and (NPs and Artificial Intelligence for Cancer Therapy) are investigated thoroughly to eliminate any bias in the study. An expanding body of literature focuses on drug delivery, drug design, bionanotechnology, and NPs for targeted delivery; however, in the context of artificial intelligence (AI) and its integration in the field of bionanotechnology, the literature exists in a broader scope, but studies covering the exact research theme as the proposed review is limited. When the search terms are narrowed to cover NPs-mediated drug delivery, including AI, there is an insufficient sample of review studies to conduct a comparative methodology or analysis. Therefore, a narrative review is considered that focuses on identifying the scope of AI and its integration in the field of bio nanotechnology, especially for targeted drug delivery, the current challenges and research gaps, and how AI can help address some of the challenges in targeted drug delivery using NPs, and improving drug efficacy. The review consists of studies from the past ten years, excluding pre-prints. In general, various review studies in the field of bionanotechnology for cancer treatment and drug delivery are considered, and some of the references are analyzed to evaluate any relevance to our research. Following the analysis of the studies,100 publications out of 150 works of literature are included, from which all the studies were evaluated to identify crticial themes such as recent applications and challenges in drug delivery, NPs as drug delivery platforms for cancer therapy, and possible scope of AI intervention to form the core of the review presented in the following sections.

### General aspects of AI in nanomedicine

AI is an umbrella term under the branch of computer science, where technologies such as machine learning, deep learning, computer vision, and natural language processing (NLP) are categorized. These technologies have the potential to allow machines to mimic human-like intelligence to perform various complex activities. Machine learning and deep learning models are trained using large and variable datasets to enable the intelligent models to predict, classify or identify patterns for a given input (15). However, it is not limited to the presented tasks as more advances in the field have led to various innovations for a wide range of real-world problems. AI algorithms can identify statistical patterns, computational intelligence, classification, prediction, and object recognition. In biomedical research, substantial growth in research for AI-enabled computational methods for drug discovery and other pharmaceutical research has been witnessed (46). AI has been influential in determining compounds, increasing productivity, ensuring regulatory compliance and transforming data, and scaling and optimizing pharmaceutical activities. In recent years, AI tools have been increasingly used for nanotechnology research in various areas, such as scanning probe microscopy, simulations, and nanocomputing. While various AI techniques exist, a specific approach called functional recognition has been used to identify the local actions from spectroscopic reactions. In particular, Artificial Neural Network (ANN) has been identified to be influential in determining the local behavior of the imaged materials, simplifying the process and allowing only appropriate variables to be considered (47).

A fundamental problem when working at a nanoscale level is the simulation. Numerical simulations are generally performed to interpret nanoscale images, as authentic optical images cannot be acquired at the nanoscale. However, various types of programs are employed for such image representations, and in many cases, it can become a complex procedure to include multiple parameters for accurate representations. In this regard, AI effectively develops simulations and more accessible interpretations of results (47).

There is an integral relationship between formulation, process factors, and controlled release in drug delivery systems which is non-linear; therefore, interconnected networks and optimization of controlled release information are essential (48). In such cases, neural network architectures are best suited as they consist of multiple layers of a node, with each layer connected, leading to prediction, classification, or recognition outcomes. The standard feed-forward neural network is the most frequently used technique in determining molecular structures, molecular fragments, topological indices, and descriptors, including studying physiochemical properties from large training sets (49). In addition, in drug design and discovery, ANNs are investigated in pharmaceutical research, understanding pre-formulations and predicting drug behaviors accurately.

### Optimizing drug discovery and drug delivery using AI

AI techniques are continuously being proposed to solve various problems in nanotechnology. The key areas are designing nanosystems, nanocomputing, and AI strategies in designing principles of nanoscale simulation, with a primary focus on reducing computational time and efficient parameter estimation, prediction, and system simulation (50). Drug delivery involves multiple approaches, such as drug formulation, manufacturing techniques, storage of bioactive compounds, and transporting to the targeted sites to ensure a higher therapeutic effect. Therefore, optimizing the drug delivery processes is essential to augment the drug's physiochemical features and overcome the instability of bioactive compounds that can severely damage the pharmacokinetic properties (51).

Recently, AI approaches have been utilized to design, characterize and manufacture drug delivery nanosystems to optimize drug delivery, where a majority of AI techniques are implemented for analyzing and interpreting biological and genetic information. This integrated approach has also been instrumental in fast-tracking the drug discovery process, identifying various functionalities of small molecules, and predicting their behaviors effectively (51). For example, various methodologies relevant to AI are proposed for predicting the efficacy of drug combinations based on drug synergies. In this context, suitable opportunities are explored for AI-based optimization for combination drug delivery using multiple classes of NPs for enhancing drug localization at the tumor site (52). In cancer therapy, physicians often work with varying genomic profiles of patients, including multiple molecular aberrations. At the same time, for drug delivery, multiple information from drug properties to biomedical parameters are considered for a patient for cancer treatment. For drugs alone, there are various molecular descriptors for medicines, which, combined with patient information comprising genetic, metabolic, proteomic, histology, and treatment history, create large datasets that make it challenging to optimize drug combinations for individual patients. This is where AI has shown promising results in the field (53).

The human body is complex as it consists of various biological membranes, each comprising physicochemical properties that separate the biological compartments. Therefore, these compartmental systems are often simplified for drug delivery. Targeted drug delivery methods must consider various factors, such as the appropriate drug administration, to ensure passage and permeation and reaching the targeted site represented by tissue or cellular membranes. In this regard, an advanced understanding of the biological environment is essential to identify biological environment interactions with the drug and its molecular features. This information complicates the predictive computation as drug delivery systems incorporate an expanding set of diversified parameters for computation (54). In order to understand the biological interactions involving membranes, AI has shown tremendous potential in pharmacokinetics evaluations with complex inputs such as drug interactions, phenotypic information, chemical interactions, and even genomic data to personalize drug delivery (55, 56). Assessing the impact of AI for optimizing drug delivery modeling, some of the critical components that are discovered are highlighted in Table 1.

**Table 1:** Impact of AI in drug development modeling.

Current AI Approaches	Focus Area	Advantage	Challenges
AI-based API system (91)	Absorption, distribution, metabolism, and excretion (ADME)	Predicting extravasation in tumor tissue. AI tools can integrate information from multiple sources and simplify the process of experimentation and in silico system simulations, resulting in enhanced drug delivery systems.	Significant challenges in the evaluation of molecular, pharmacokinetics, and patient information.
Passive AI (91)	Molecular entity features of the drugs	These AI models can help include new molecular entity features, including commonly known molecules, for predicting effective treatment based on a molecule's bioavailability or local concentration for better therapeutic outcomes.	The availability of diverse datasets provides information on various molecules as it is considered in the range of 10^60^ molecules. If such datasets are not available, AI models will provide biased information.
Deep Neural Networks (DNNs)	Drug repurposing	DNNs could efficiently classify complex drug action mechanisms based on the pathway level. Further, the model could classify drugs into different categories, such as functional, therapeutic, and toxicity (92).	According to the authors, there were instances of misclassified samples that could indicate novel use of a particular drug or discoveries. However, beyond a certain level of misclassification, it may impact the accuracy of the proposed model.
Generative Adversarial Network (GAN)	De Novo generation of new molecules with suitable molecular properties in silico	GANs provide the ability to generate molecular fingerprints with predefined anticancer properties. This model also efficiently processes sizeable molecular data sets, allowing drug development of new molecules for cancer treatment (93).	The author suggests that further studies are required to understand the reconstruction aspect that occurs from generated samples. Another critical area of this model is the need for high computational power.
Feed-Forward Multi-Layer Perceptron	Predicting drug sensitivity using cell line screening data to identify dose response.	The model could predict cancer cell sensitivity to drug molecules with features derived from cells and drugs comprising genomic features and chemical information, respectively (56).	The model could not capture all the information related to gene-to-drug associations since the dataset did not provide more considerable drug sensitivity information. There is a scope for improvement as extended input features such as basal transcriptional profiles and phosphoproteomic can be included to enhance the model's predictive capability.
Artificial Neural Network (ANN)	Predicting the synergy of anticancer drugs.	ANN with a back-propagation method was implemented to predict and quantify the synergism of anticancer drugs. The results helped determine the drug concentrations and cytotoxicity (94).	Although the model performed well in determining optimal compositions and presented the maximum synergistic effect, the model's capability with more complex information could be evaluated.
Deep Synergy (A Deep Learning Feed Forward Neural Network)	Predicting anticancer drug synergy	Drug synergy prediction based on chemical and genomic information. The purpose is to accurately predict the synergy of the drug combination and compare the methods while distinguishing different cancer cell lines (95).	The model performed marginally better than other machine learning methods. However, there is a scope for improvement in accuracy as the dataset had limited information on the number of different drugs and cell lines considered for experimentation.

### Patient biomarker detection and profiling for targeted drug delivery

Cancer biomarkers play a crucial role in oncology as critical information can be obtained for risk assessments, determining prognosis, identifying the response to treatment, diagnosing the disease, and monitoring the disease progression effectively(57). According to National Cancer Institute, every cancer patient has unique biomarker patterns, which help to understand how specific cancer treatment may work. Therefore, obtaining biomarker information helps clinicians to choose the best cancer treatment for patients (58). Moreover, the biomarkers differentiate in patients; therefore, identifying the distinct molecular signature of the patient helps in screening cancer patients for cancer therapy. In addition, these patients can be classified according to the stage of cancer, where the tumor could be either in a localized stage or has metastasis (59).

Recently, a wide range of biomarker testing has been performed to help select the most appropriate cancer treatment for the patient. Most biomarker tests include identifying genetic markers, proteins, or other tumor markers. However, biomarker testing includes checks for single and multiple biomarkers, also known as multigene testing. For example, a standard cancer biomarker test performed for breast cancer patients is the Oncotype DX test, performed to identify the activities of 21 genes for predicting whether chemotherapy is likely to work for the particular patient (58). These prediction necessities are the areas that could be further improved with the integration of prediction and classification AI models.

At the same time, the primary factor to be considered when discussing drug delivery, especially for cancer therapeutics, is to deliver the targeted drugs accurately, where it activates only in the targeted tumor site without harming any healthy tissues near the organ of interest (15). In addition, drug-loaded NPs coated with ligands, antibodies, or other cellular markers allow them to bind to the target cells, resulting in improved treatment response. However, precision in cancer treatment is achievable if molecular profiles for each patient are obtained. These may include disease-related information and biomarkers. Thus, disease profiles based on omics data provide highly essential genomic, proteomic, metabolomics, microbiomic, and epigenomic information (15).

Many nanomaterials, such as quantum dots, gold NPs, and carbon nanotubes, are implemented for biomarker sensing applications. In light of the recent developments, nanomaterials are the most promising technologies in cancer biomarker sensing, capable of high sensitivity and specificity. The utility of nanomaterials is currently investigated for both *in vivo* and *in vitro* medical applications to improve diagnosis and therapeutic platforms. In addition, these nanomaterials have opened new avenues for attaining precise diagnostic information that can help in the early diagnosis of disease, identifying the prognosis of the disease, and personalizing treatment plans (60).

NPs are versatile candidates widely implemented for biomedical applications, including their uses for radiotherapy enhancement, drug delivery, and diagnostic assays (61). In addition, novel NPs-based systems are explored for better penetration of cancer drugs and tracking within the body to enable efficient cancer therapy with reduced risks compared to conventional approaches (62). The unique range of characteristics of NPs allows for easier synthesis and functionalization for cancer therapeutics. Moreover, metal NPs such as gold and silver also offer tunable properties to desirable sizes and compositions for accurate imaging of the tumor sites, which allows for improving targeted drug delivery methods. Most recently, researchers have highlighted the possibility of functionalizing NPs with antibodies and peptides or DNA and RNA for effecting targeting of cancer cells while using biocompatible polymers such as polyethylene glycol for prolonged *in vivo* circulation for drug delivery applications (63).

#### Existing challenges in cancer imaging that can be addressed using AI and NPs

High-quality cancer imaging necessitates multifaceted, disease-specific information, including examinations, image post-processing, and interpretation for clinical reporting and treatment planning. The imaging procedures focus on identifying tumor entities and metastatic patterns and acquiring supplementary clinical information from various imaging modalities to assess tumor response to therapies and identify complications and possibilities of tumor recurrence (64).

Therefore, recent developments have focused on the combination of molecular imaging and drug delivery for visually assessing the drug delivery process in real-time and understanding the therapeutic agents' *in vivo* efficacy. Researchers aim to build a multifunctional theranostic probe for targeted drug delivery that can help in molecular imaging and controlled drug release for optimal therapeutic response. In addition, conventional methods face the challenges of evaluating pharmacokinetics and pharmacodynamics information, but molecular imaging decreases the workload, generating more precise data for developing candidate drugs with optimal target specificity and pharmacodynamics efficacy, respectively (65).

Medical imaging is vital in evaluating nanomedicine-based drug delivery systems, but exploring these avenues has been limited. Integrating imaging in drug delivery aims to gain valuable information on pharmacokinetics, biodistribution, and accumulation of drugs on the targeted tumor site. These imaging outcomes provide evidence on uptake levels of nanomedicine on the tumor site to confirm the permeability and retention effect (EPR), which has high levels of variability in cancer patients (66). From a developmental perspective of improving drug delivery systems, imaging answers some of the critical questions on where the drugs go inside the body, their circulation time, how they clear from the body, if the drugs are reaching the target, and whether the drugs are getting released appropriately (67).

For example, Malvern Panalytical has developed a nanoparticle tracking analysis (NTA) system to visualize and measure different sizes and drug concentrations. This software calculates hydrodynamic diameters with fluorescence modes to define labeled particles. In addition, real-time monitoring of the nanoparticle population can be characterized and validated in a simplified manner (68). In this context, it can be suggested that if a specific software can be programmed to achieve the success of NTA systems, there is also scope for AI to enhance nanoparticle tracking systems for improving analysis, processing, and delivering high-resolution images with appropriate labels. A recent study has revealed that characterizing the nanoparticles is critical for medical diagnosis and nanoparticle-mediated drug delivery. Furthermore, the author suggests that most biological applications have shorter trajectories. Hence, to achieve a label-free method for quantifying the sizes of nanoparticles and providing the subwavelength particles simultaneously, a convolutional neural network (CNN) was developed to analyze holographic images of single particles while using the acquired information to successfully distinguish and quantify the size of the particle, including its reflective index accurately (69).

**Figure 3:**
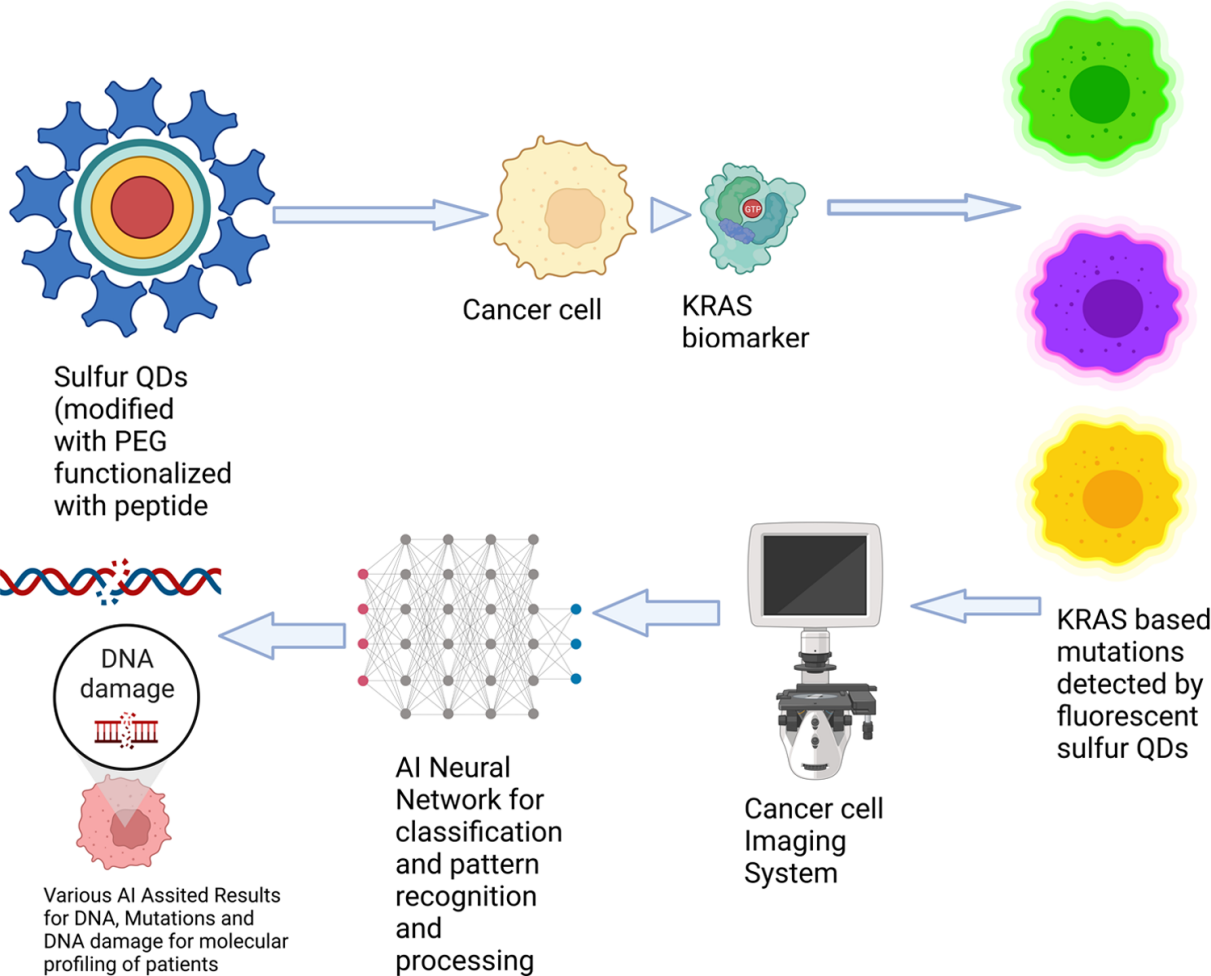
Graphical representation of a potential AI-enabled biomarker sensing approach. (Reprinted from “Nanoparticle-Mediated Targeted Drug Delivery to Cancer Stem Cells”, by BioRender, June 2020).

However, to achieve a well-optimized drug delivery system, evaluating pharmacodynamics and pharmacokinetics properties is essential for achieving a deliverable formulation and increasing drug efficacy. Due to the highly sensitive characteristics of the free drugs, radionuclide imaging is implemented to assess the appropriate drug delivery vehicle (65). Clinical studies indicate that in the field of image-guided drug delivery, the most frequently used imaging modalities are fluorodeoxyglucose positron emission tomography (FDG PET), Computed Tomography (CT), and single photon emission computed tomography (SPECT) to monitor the response of the treatment (66). However, radiotracers like fluorodeoxyglucose (F-FDG 18), commonly used for cancer diagnosis, have a relatively shorter shelf life due to the physical decay of ^18^F with a half-life of 109 min (71). Although this time duration is sufficient for PET scanning, NPs with fluorescent properties like quantum dots may provide a longer retention time in tumor tissues and produce higher concentrations than in other tissues (71). Therefore, this approach may help improve diagnosis as variable FDG uptakes often result in noise in diagnostic images. Combined with AI, these results can help improve drug delivery molecular imaging (refer to Figure 3).

While targeted drug delivery is a significant advancement in cancer therapy, its success can be ensured when biomarker-sensing NPs can facilitate early disease detection and provide prognostic information. Recent research highlights that prognostic information on the Kirsten Rat Sarcoma Virus (KRAS) mutation is critical for patient survival. It has also been identified that the most prolonged survival rate of patients with KRAS mutation at 36 months was only 24 percent (72). In this case, integrating artificial intelligence (AI) helps evaluate all the complex heterogeneous tumor information and patterns acquired from biomarker sensing NPs to devise an accurate treatment plan and aid in the faster detection of mutations, classifying mutated and non-mutated biomarkers, better drug and protein interaction for greater drug efficacy and improved drug delivery.

For example, in the case of lung cancer, a particular biomarker that is essential to be identified is the KRAS gene, especially the KRAS G12C, accounting for nearly 44% of all KRAS mutations (73). At the same time, the KRAS mutations are associated with poor response to standard therapies (74). Therefore, these cancer genes can potentially convert normal cells to cancerous ones. Moreover, each KRAS mutant variant has a distinct profile; therefore, creating a patient-based molecular profile is essential before targeted drug delivery (73). Consequently, researchers have investigated the potential of quantum dots to distinguish the KRAS gene in its non-mutated form from the cancerous KRAS. In general, cancerous cells overexpress a receptor on the cell membrane, which can be effectively targeted with the help of a fluorescent probe such as the quantum dot (75). When fluorescence quantum dots are implemented, they produce variable lights of different colors, which can help accurately identify cancer cells and their gene signatures. The resultant images can be further processed with AI for tumor location identification and planning drug delivery. In addition, AI-enabled approaches also help in drug targeting and ratiometric delivery.

Similarly, classification models can be used to create molecular profiles for patients depending on the patient response and drug synergy to determine drug efficacy, whereas predictive models can help determine the prognosis of the disease based on the acquired information. AI algorithms have proven capabilities in processing large datasets with complex patterns, and as such, implementing AI for biomarker imaging can be exploited to improve the diagnosis and treatment of cancer. In addition, using AI to predict the NPs-based interaction with the target drug, tumor site, and cell membranes can provide supplementary information on drug encapsulation and drug release kinetics, optimizing nanomedicine formulation for cancer treatment (15, 16).

Nanosensors comprise electrochemical and mechanical properties that improve the signal-to-noise ratio in biomarker sensing, including the ability to detect a low concentration of molecular signatures in the tumor microenvironment. Therefore, AI can help detect multiple biomarkers that can help construct the unique disease signature using high computational analysis for accurate diagnosis (15, 76). For computation and targeting biomarkers for drug delivery, Principal component analysis (PCA) and neural networks have been proposed to increase the accuracy of classifying the signals from electronic nose applications. In contrast, clustering algorithms have been effective in pattern recognition necessities to identify inherent variations in disease information between patient populations (15).

### Drug delivery using intelligent AI solutions and nanorobots

Researchers have focused on developing and deploying micro robotics in medicine in the past several years. These nanomachines focus on several medical tasks, such as drug delivery *in situ*, targeting disease cell membranes, and even performing micro surgeries. Although persistent challenge arises in terms of material design, production, availability, and biocompatibility, studies indicate the tremendous potential of nanorobots (77). A recent study emphasized using mesoporous silica NPs containing unease enzymes and gold NPs as nanomotors. These nanomotors were radiolabeled for *in vivo* imaging. In addition, PET was implemented for quantitatively tracking nanomotors, thereby improving real-time imaging and tracking of active swarming dynamics and paving the way for theranostic applications in drug delivery (78). Due to remarkable advancements in engineering and bio nanotechnology, increasing developments are witnessed in integrating smart sensors, power supply, and AI in the nanorobots (79). Researchers are also investigating the feasibility of automation in molecular manufacturing, wherein AI technologies can control the behavior and motion of the nanorobots (80).

Similarly, AI-based simulations and modeling may help devise better nanorobots for controlled drug delivery with effective nano communication (81). Novel technologies DNA nanorobots are developed for drug delivery and biosensing. However, ANNs are considered critical components of these nanorobots as the neural network enhances prediction capabilities and optimizes their performance for detecting tumor cells for targeted drug delivery (46, 82). While the significant impact of AI on nanorobots has been highlighted, fuzzy logic is another approach that has been identified to be effective in estimating drug dosage for intracellular delivery following the tumor diagnosis. In particular, fuzzy models can effectively provide the linear mapping required for identifying the appropriate dose for intracellular delivery (46, 83).

## Future outlook and challenges of nanoparticle mediated drug delivery using novel technologies and AI

The continuous advances in technology have led to state-of-the-art techniques, such as AI, that have been promising in the field of drug discovery and optimization. However, nanomedicine has various challenges, despite boasting numerous advantages. The most widely highlighted challenges are the EPR effect, the size and nature of drug delivery systems, drug reservoir design issues, biocompatibility, drug concentrations, and toxicity, among various issues (15, 46, 84). Another critical aspect is the fabrication challenges when focusing on nanorobots as future theranostic applications (85). However, challenges associated with nanorobots for drug delivery are noise and unknown parameters that may cause failure in appropriate drug dosage delivery and the inability to differentiate healthy cells and tumor cells (46, 86). AI has the potential to solve several challenges related to nanotechnologies, such as data analysis and processing of complex data and facilitating during drug discovery and drug design, respectively. In addition, this form of AI integration allows for addressing the limitations with accurate dose delivery.

Moreover, AI is highly effective in genetic programming, providing supplementary information on cancer genomics by identifying various complex patterns. ANNs, fuzzy logic, and decision trees are critical components in modern drug discovery processes (65, 66).

While ANNs are highly effective for classification, pattern recognition, and prediction, other networks such as supervised associating networks, can be of immense value, especially for response surface methodology to enable faster investigation of relationships between explanatory and response variables (87). At the same time, fuzzy logic-based drug delivery systems have been identified to provide faster response times to achieve effective regulation and automation for drug delivery scheduling and managing arterial and venous circulation using pharmacological agents (88). Another state-of-the-art AI solution that has garnered interest in silico medicine is reinforcement learning. The primary reason is these AI algorithms' ability to learn from their environment and be less dependent on datasets. Recently, a study proposed a supervised learning algorithm capable of identifying missing features from the datasets and detecting the differences between normal and diseased patient profiles (89, 90).

Incorporating AI in nanotechnology and pharmaceutical research has significantly reduced the time and cost involved in drug discoveries, assessing pharmacodynamics and pharmacokinetic profiles of various drugs and reducing false favorable rates. However, challenges arise in high computational power, availability, maintenance, ethical issues, and reliability of AI-enabled outcomes (46). Meanwhile, suppose AI is considered for nanomedicine. In that case, challenges like overfitting, validation, and bias have to be addressed to ensure robust models are available for the prediction of drug synergies, identifying appropriate molecular combinations, and biomarker imaging and helping in accurate drug delivery and drug efficacy. Similarly, a common problem is the availability of large datasets with multiple sets of clinical information to train AI models and optimize for accurate drug delivery. Currently, there is a lack of AI utilization for drug development and delivery, but in recent years, pharmaceutical industries and bioinformatics are some of the upcoming areas where successful AI integration has been keenly observed. Despite the challenges, it is noted by researchers that AI is speeding up drug development, including real-world experiments. In addition, AI is also investigated for gene therapies and contributing towards regenerative medicine. Similarly, AI has been limited in drug delivery, but there is promise in its contribution toward future therapeutic applications to enhance drug delivery (90).

## Conclusion

The field of nanomedicine is rapidly evolving, and versatile drug carriers are being evaluated for their effectiveness in reaching the targeted tumor sites and improving localized drug delivery. Furthermore, combining nanotherapies with hybrid approaches is continuously evaluated to enhance treatment efficacy for cancer patients. However, these approaches are confronted by various challenges of conventional drug development and delivery methods. While it is essential to understand drug synergies, identifying individual patient profiles based on their unique molecular signatures has become critical in ensuring the ultimate success of targeted drug delivery. These are necessities because the rate of treatment failures and lack of response to treatments continue to remain high. At the same time, to improve cancer treatments, more clinical parameters are required to minimize treatment failures and chances of cancer reccurrence. In this context, intelligent computational models can process complex data and produce accurate results successfully. Therefore, AI plays a crucial role in devising a roadmap to assess real-time monitoring of drug delivery procedures, classifying patients according to molecular signatures, and also provide actionable insights on treatment response, ability to quantify clinical information, and, most importantly, contributing towards image-guided drug delivery due to proven capabilities with clinical imaging. Although several techniques have been evaluated in pharmaceutical and nanotechnology domains over the years, the studies focusing on AI for targeted drug delivery have remained limited. Therefore, this study aimed to provide a pathway on how the integration of AI can help overcome some of the limitations of fabrication techniques and the likely impact on nanoparticle imaging, patient profiling, and cancer biomarker detection to help enhance the outcomes of nanotechnology-based therapeutics. Future works may lead to building intelligent solutions for biomarker detection and nanoparticle tracking and analysis systems by taking critical insights from the survey.

## Data Availability

The original contributions presented in the study are included in the article/Supplementary Materials, further inquiries can be directed to the corresponding author.
